# CCR6+ group 3 innate lymphoid cells accumulate in inflamed joints in rheumatoid arthritis and produce Th17 cytokines

**DOI:** 10.1186/s13075-019-1984-x

**Published:** 2019-08-30

**Authors:** Ayako Takaki-Kuwahara, Yojiro Arinobu, Kohta Miyawaki, Hisakata Yamada, Hirofumi Tsuzuki, Kensuke Irino, Masahiro Ayano, Yasutaka Kimoto, Hiroki Mitoma, Mitsuteru Akahoshi, Hiroshi Tsukamoto, Takahiko Horiuchi, Hiroaki Niiro, Koichi Akashi

**Affiliations:** 1 Department of Medicine and Biosystemic Science, Kyushu University Graduate School of Medical Sciences, 3-1-1 Maidashi, Higashi-ku, Fukuoka, 812-8582 Japan; 2 Division of Host Defense, Medical Institute of Bioregulation, Kyushu University, Fukuoka, Japan; 3 Department of Stem Cell Biology and Medicine Faculty of Medical Sciences, Kyushu University, Fukuoka, Japan; 4 Department of Internal Medicine, Kyushu University Beppu Hospital, Beppu, Japan; 5 Faculty of Medical Sciences Medical Education, Kyushu University, Fukuoka, Japan

**Keywords:** Innate lymphoid cells, Rheumatoid arthritis, CCR6, Th17 cytokines

## Abstract

**Background:**

Recent studies show that innate lymphoid cells (ILCs) contribute to the development of chronic inflammation and autoimmune disease. In this study, we assessed the ILC function in the development of rheumatoid arthritis (RA).

**Methods:**

In a mouse model of collagen-induced arthritis (CIA), we identified and purified the ILC subsets in peripheral blood (PB), local lymph nodes (LNs), and joints by fluorescence-activated cell sorting and used quantitative PCR to assess the expression levels of representative cytokines. We also correlated the frequencies of each ILC subset in synovial fluid (SF) with clinical parameters in RA patients.

**Results:**

In the CIA model, the proportion of CCR6+ ILC3s to total ILCs in joints with active inflammation significantly increased relative to non-arthritic joints (median 29.6% vs 16.7%, *p* = 0.035). CCR6+ ILC3s from mice with arthritis expressed significantly higher levels of IL-17A and IL-22 mRNA than did comparable cells from control mice (*p* < 0.0001 and *p* = 0.015). In RA patients, the proportion of CCR6+ ILCs in SF was positively correlated with tender joint counts (TJC) and swollen joint counts (SJC) (*ρ*=0.689, *p* = 0.0032 and *ρ*=0.644, *p* = 0.0071, respectively). Levels of CC chemokine ligand 20 (CCL20) increased in SF of patients with RA and were significantly correlated with CCR6+ ILC number (*ρ*=0.697, *p* = 0.0001).

**Conclusion:**

CCR6+ ILC3s may play some roles in the development of RA through the production of IL-17 and IL-22.

**Electronic supplementary material:**

The online version of this article (10.1186/s13075-019-1984-x) contains supplementary material, which is available to authorized users.

## Background

Innate lymphoid cells (ILCs) are recently discovered immune cells that do not express rearranged antigen receptors and do not have antigen specificity [1]. ILCs represent the innate immune system’s counterpart to helper T cells and are classified into three subsets, group 1 (ILC1s), group 2 (ILC2s), and group 3 (ILC3s), based on the patterns of cytokine production and transcription factor expression [1]. Accordingly, ILC1s require T-box transcription factor (T-bet) for differentiation and produce interferon-γ (IFN-γ); ILC2s express the highest levels of GATA-binding protein 3 (GATA-3) and produce interleukin-4 (IL-4), IL-5, IL-9, and IL-13; and ILC3s express and are regulated by retinoic acid receptor-related orphan receptor γt (RORγt) and produce IL-17 and IL-22. ILC3s are further subdivided based on the expression of either CC chemokine receptor 6 (CCR6) or natural cytotoxicity receptor (NCR), which is constituted by NKp46, NKp44, and NKp30 and is expressed mainly on natural killer (NK) cells [2, 3]. Increasing evidence shows that ILCs contribute to chronic inflammation and autoimmune disease [4–6]. There are some reports that show ILC3s are the key population in inflammation of spondyloarthritis (SpA) [7–10]; on the other hand, a limited number of studies have addressed the role of ILCs in rheumatoid arthritis (RA), and it is controversial which subset is important in RA development. Recent studies revealed that ILC2s are essential for the resolution of inflammation in RA [11, 12], although another group showed that GM-CSF-producing ILC2s play a role in the development of arthritis [13]. In contrast, ILC1-like and ILC3-like cells have been shown to promote inflammation in RA [14–16]. ILC3s produce IL-17 and IL-22, cytokines reportedly essential for RA pathogenesis [7, 8, 17]. Furthermore, *CCR6* has been identified as an RA disease susceptibility gene [18]. These findings suggest overall that CCR6+ ILC3s function in RA development.

In this study, we evaluated the number, distribution, and function of ILC1, ILC2, and ILC3 cells in a murine arthritis model and in patients with RA. Our results suggest that CCR6+ ILC3s play a crucial role in RA development.

## Materials and methods

### Mice

Male C57BL/6J mice were evaluated at 11–12 weeks of age. All mice used in this experiment were purchased from Charles River Japan Inc. (Yokohama, Japan) and maintained under specific pathogen-free conditions at the Kyushu University Animal Facility. All animal experiments were approved by the Animal Care and Use Committee, Kyushu University.

### Induction of collagen-induced arthritis

Mice were immunized subcutaneously at the base of the tail with 4 mg/ml chicken type II collagen (CII) (Collagen Research Center, Tokyo, Japan) emulsified with an equal volume of complete Freund’s adjuvant (CFA) containing *Mycobacterium tuberculosis* H37Ra at a concentration of 5 mg/ml (Difco, Detroit, MI, USA). Mice were boosted on day 21 with CII emulsified with CFA containing *Mycobacterium tuberculosis* H37RA at the same concentration.

### Measurement of serum anti-type II collagen antibody levels

Serum levels of anti-CII antibodies were measured by enzyme-linked immunosorbent assay (ELISA). Microtiter plates were coated with chicken CII (10 μg/ml) overnight at 4 °C. After washing and blocking, serum samples were added in serial dilutions and incubated 2 h at room temperature. After four washes, HRP-conjugated anti-mouse IgG antibody (Leinco Technologies Inc., St. Louis, MO, USA) was added and incubated for an hour at room temperature. Antibody binding was visualized using TMB substrate (Seracare Life Sciences Inc., Milford, MA, USA). All plates were read at 450 nm using MultiSkan FC (Thermo Fisher Scientific KK, Yokohama, Japan).

### Flow cytometric analysis of mouse ILC subsets

To obtain a cell suspension from mouse joints, the entire legs were dissected and the muscles and tendons were removed. To avoid contamination with the bone marrow, the femur was disarticulated by pulling the femoral head. The ligaments and tendons around the joints were cut a few millimeters. The knee and ankle joint were then opened. The legs were incubated in a mixture of enzymes containing 100 U/ml collagenase (Wako Pure Chemical Industries, Ltd., Osaka, Japan) and 100 μg/ml DNaseI (Sigma-Aldrich, St. Louis, MO, USA) and shaken for 60 min at 37 °C. After digestion, the legs were removed, and cells were purified using Percoll density centrifugation, filtered, and counted using automated cell counter.

The popliteal lymph nodes and spleen were mechanically mashed and filtered. Splenocytes and peripheral blood cells were hemolyzed with NH_4_Cl and washed with PBS. For surface staining, single-cell suspensions of joint cells, lymph node cells, splenocytes, and peripheral blood cells were washed with cell staining buffer and stained with a cocktail of antibodies for 30 min at 4 °C. ILCs were defined as CD45.2 positive, lineage (CD3, CD8, CD19, B220, Ter119, Ly-6G, CD11c, CD11b) negative, CD90.2 (Thy1.2) positive, and CD127 (IL-7R) positive. ILCs were subsequently classified as ILC1s, ILC2s, CCR6+ ILC3s, or NKp46 (CD335)-positive ILCs (NKp46+ ILCs), based on the expression of NK1.1, ST2 (the receptor for IL-33), CCR6, and NKp46.

### RNA extraction and quantitative real-time PCR

Each target population was sorted directly into ISOGEN II (Nippon Gene Co., Tokyo, Japan), and total RNA was extracted and reverse transcribed using the SuperScript III First-Strand Synthesis System (Invitrogen). Quantitative real-time PCR was conducted with a 7500 Real-Time PCR System (Applied Biosystems, Foster, CA, USA) or an Mx 3000P Real-Time PCR System (Agilent Technologies, Santa Clara, CA, USA). TaqMan probes for Tbx21 (Mm00450960_m1), Gata3 (Mm00484683_m1), RORC (Mm01261022_m1), Ifng (Mm01168134_m1), Il5 (Mm00439646_m1), Il13 (Mm99999190_m1), Il17Aa (Mm00439618_m1), and Il22 (Mm00444241_m1) were all purchased from Applied Biosystems. 18S rRNA expression served as the internal control.

Relative transcript levels were calculated using the ddCT method.

### Patients

Patients with RA met the 1987 American College of Rheumatology classification criteria. Twenty-three synovial fluid (SF) samples were obtained from 17 patients with RA. Patient details are provided in Additional file 8: Table S2. Twenty-four SF samples of patients with osteoarthritis (OA) were obtained from 20 patients. Informed consent was obtained from all subjects in accordance with the Declaration of Helsinki. The Institutional Review Board of Kyushu University Hospital approved all research on human subjects.

### Flow cytometric analysis of ILCs in synovial fluid in RA patients

Mononuclear cells were isolated from SF using density gradient centrifugation with LSM (MP Biomedicals, LLC, Santa Ana, CA, USA). ILCs were defined as single cells within the lymphocyte gate on the scatter plot that were CD45 positive, lineage (CD3, CD19, CD16, CD94, CD14, CD1c, CD11b, CD11c, CD235ab, FcεRI, CD34) negative, and CD127 (IL-7R) positive. ILC subsets were classified based on the expression of CD117 (c-Kit), chemoattractant receptor homologous molecule expressed on Th2 cells (CRTH2) (CD294), and NKp44 (CD336). Specifically, ILC2s were defined as CRTH2 positive, and other subsets were CRTH2 negative. ILC1s, NKp44− ILC3s, and NKp44+ ILC3s were defined as CD117− NKp44−, CD117+ NKp44−, and CD117+ NKp44+, respectively. CCR6+ ILCs were defined as CCR6-positive cells in total ILCs. Antibodies used in flow cytometric analysis are listed in Additional file 7: Table S1 and were purchased from BD Biosciences (San Jose, CA, USA), BioLegend (San Diego, CA, USA), Beckman Coulter (Villepinte, France), or MD Bioproducts (Saint Paul, MN, USA). Cells were analyzed with a FACS Aria III system (BD Biosciences). FlowJo software (Tree Star Inc., Ashland, OR, USA) was used to analyze flow cytometric data.

### Chemokine measurement

SF from RA and OA patients was centrifuged, and supernatants were collected and stored at − 80 °C. Human CC chemokine ligand 20 (CCL20) in SF was measured by ELISA using a Human CCL20 ELISA kit (BioLegend) with a detection limit of 2.9 pg/ml, according to the manufacturer’s instructions.

### Statistical analysis

Statistical comparisons between the two groups were performed using Student’s *t* test or the Wilcoxon rank-sum test according to the distributions. Results are expressed as means ± SD or the median (range). Differences between the three groups were assessed by the Kruskal-Wallis test followed by the Wilcoxon rank-sum test. Spearman’s rank correlation was performed to correlate the proportion of ILC subsets in SF with clinical parameters of RA patients. *P* values < 0.05 were considered significant. All analyses were performed using JMP statistical software version 13.0 (SAS Institute, Cary, NC, USA).

## Results

### Identification of ILC1s, ILC2s, and ILC3s in wild-type mice

**Fig. 1:**
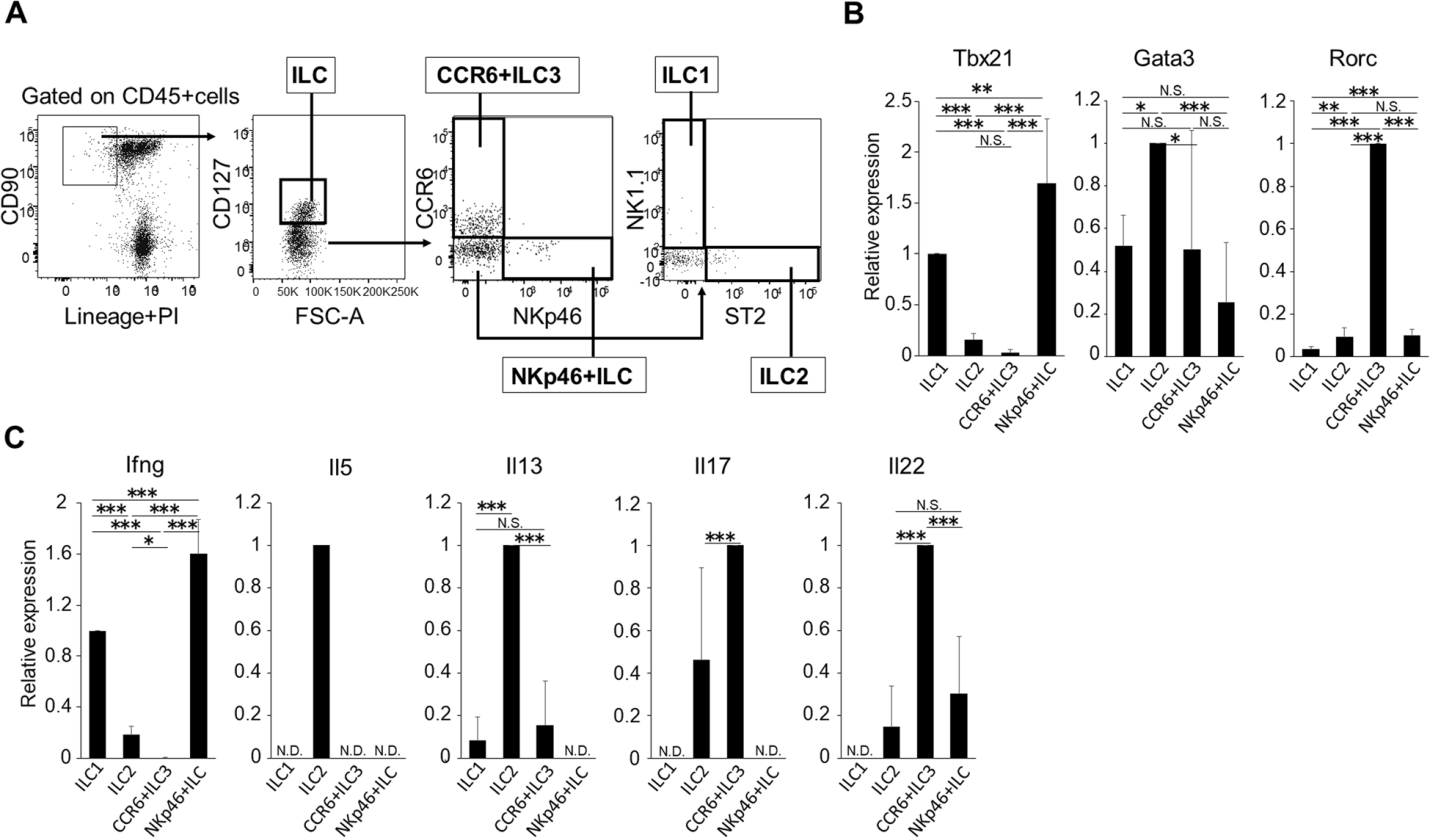
Expression of lineage-associated transcription factors and cytokines in ILC subsets from wild-type mice. **a** Gating strategy for ILC1s (NK1.1+), ILC2s (ST2+), CCR6+ ILC3s (CCR6+), and NKp46+ ILCs (NKp46+). ILCs were defined as CD45+, Lineage−, CD90+, and CD127+. **b**, **c** Quantitative real-time-PCR analysis of lineage-affiliated transcription factors (**b**) and cytokines (**c**) in purified ILC subpopulations. Each ILC subset was obtained from the spleen cells of two to three mice. Results are expressed as means ± SD in triplicate samples from two independent experiments, each using two to three mice, and were evaluated using an unpaired Student’s *t* test. N.S., not significant; N.D., not detected

We identified ILC subsets in splenocytes of adult male C57BL/6J mice. Identification of ILC1s, ILC2s, and ILC3s by surface markers [1, 19] is described in the “Materials and methods” section (Fig. 1a). ILCs were isolated as cells negative for lineage markers and positive for CD45, CD90, and CD127. ILCs were subsequently classified as CCR6+ ILC3s or NKp46+ ILCs. In cells negative for both CCR6 and NKp46, ILC1s and ILC2s were defined based on the expression of NK1.1 and ST2, respectively. In our gating strategy, NKp46+ ILCs include a part of ILC1s and NCR-positive ILC3s, as NKp46 is expressed on the surface of both. Because most NK cells express lineage marker CD11b and do not express CD127 [20], the majority of NK cells should not be included in ILC fraction.

We next confirmed the identity of each ILC subset using quantitative real-time PCR analyses to detect representative transcription factors (TFs) and cytokines in purified subpopulations (Fig. 1b, c). Expression levels of T-box 21 (Tbx21) and IFN-γ mRNA increased in both ILC1s and NKp46+ ILCs. ILC2s expressed the highest level of GATA3 mRNA, while other ILCs expressed more moderate levels. IL-5 and IL-13 mRNAs were highly expressed in ILC2s, and CCR6+ ILC3s showed increased transcript levels of RAR-related orphan receptor C (RORC), IL-17A, and IL-22. These data indicate successful fractionation of ILC subsets.

### CCR6+ ILC3 populations expand in the joints of arthritic mice

**Fig. 2:**
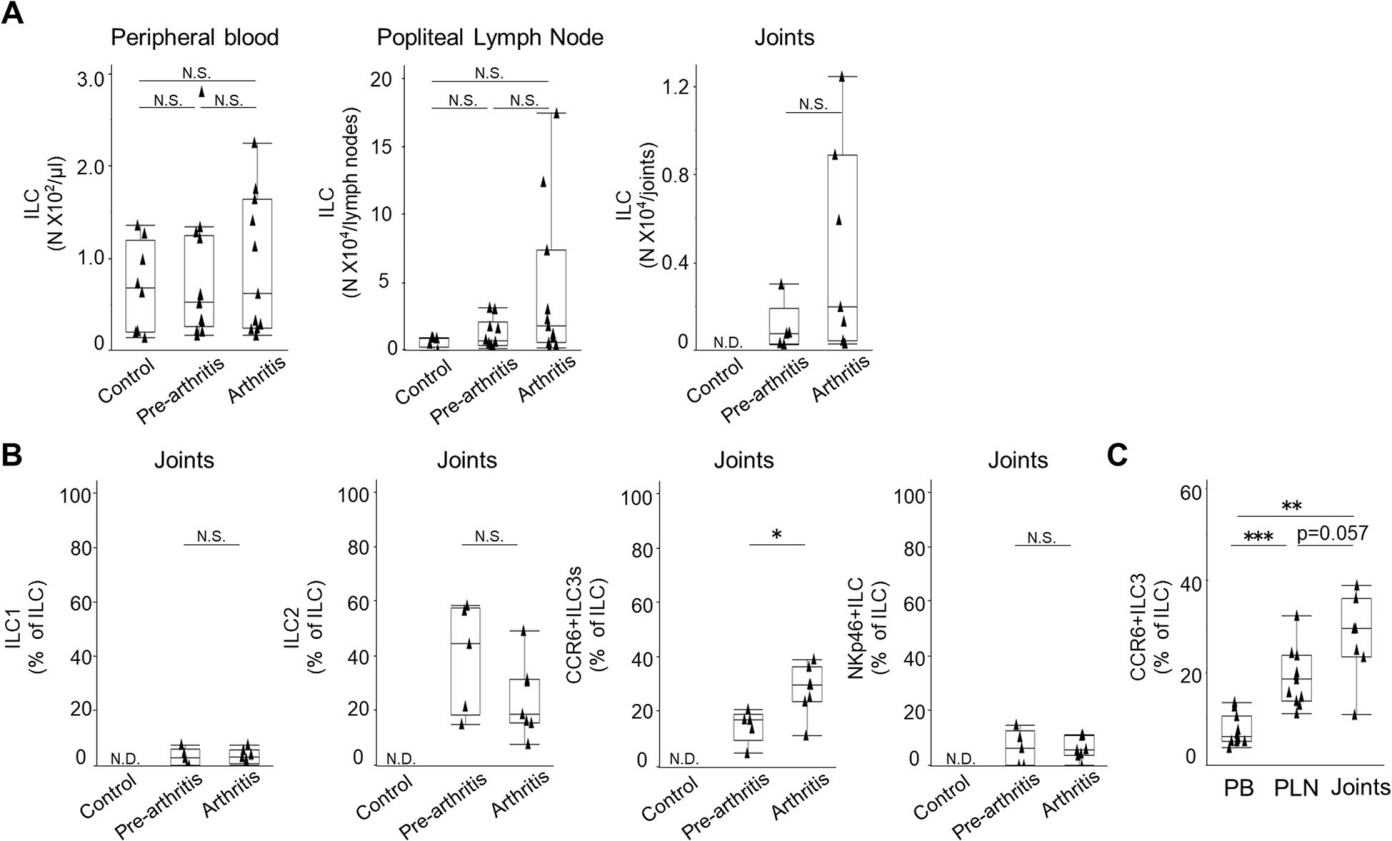
CCR6+ ILC3s accumulate in the inflamed joints in mice. **a** (Left) absolute number of ILCs in peripheral blood of control mice (*n* = 8), pre-arthritic mice (*n* = 13), and mice with arthritis (*n* = 11) (Kruskal-Wallis test; *p* = 0.7605). (Middle) absolute number of ILCs in popliteal lymph nodes of control mice (*n* = 5), pre-arthritic mice (*n* = 10), and mice with arthritis (*n* = 11) (Kruskal-Wallis test; *p* = 0.1708). (Right) absolute number of ILCs in joints of pre-arthritic mice (*n* = 5) and mice with arthritis (*n* = 7). **b** Comparison of the proportion of each ILC subset among total ILCs in the joints of pre-arthritic (*n* = 5) and arthritic (*n* = 7) mice. The proportion of CCR6+ ILC3s in the joints of arthritic mice was greater than that seen in pre-arthritic mice. Data are expressed as a percentage of total ILCs. **c** The proportion of CCR6+ ILC3s in the peripheral blood, popliteal lymph nodes, and joints of arthritic mice. The proportion of CCR6+ ILC3s increased in the joints compared to peripheral blood or popliteal lymph nodes (Kruskal-Wallis test; *p* < 0.0001). In all three panels, data points in graphs represent individual mice. Results are expressed as the median and interquartile range based on the Wilcoxon rank-sum test. (*p < 0.05, **p < 0.01, ***p < 0.001). N.S., not significant; N.D., not detected; PB, peripheral blood; PLNs, popliteal lymph nodes

We next evaluated the ILC subsets in PB, popliteal lymph nodes (PLNs), and joints isolated from collagen-induced arthritis (CIA) mice. Following CIA induction, mice exhibited both arthritic and non-arthritic phenotypes. However, although the latter group did not exhibit evidence of joint swelling, we detected anti-CII antibodies in their sera (Additional file 1: Figure S1), suggesting that these mice represent a preclinical phase of RA development. Thus, we analyzed these mice as “pre-arthritic mice.” RA synovitis is initiated by an influx of leukocytes from the PB to the synovial tissue. Therefore, we asked whether the number of ILCs increased in inflamed joints of CIA mice, and if so, which subset accumulated in the joints. To do so, we first counted the absolute number of ILCs in the PB, PLNs, and joints of pre-arthritic, arthritic, and wild-type mice as control (Fig. 2a). We observed comparable numbers of ILCs in PB of mice in all three conditions. The number of ILCs in the PLNs of arthritic mice was higher than seen in control mice, although that increase was not statistically significant (median, 1.8 × 10^4^/lymph node vs 8.8 × 10^3^/lymph node, *p* = 0.089). ILC numbers increased in the joints of arthritic mice but were minimal in the joints of control mice. In short, the number of ILCs increased in local lymph nodes and joints of arthritic mice, although differences were not statistically significant. This result suggests that ILCs accumulate at the inflammatory sites.

We next calculated the ratios of ILC subsets to total ILCs in the joints of pre-arthritic and arthritic mice, while ILC number in the joints of control mice was too minimal to be analyzed. In arthritic mice, the proportion of CCR6+ ILC3s to total ILCs increased significantly compared to that seen in the joints of pre-arthritic mice (median, 29.6% vs 16.7% *p* = 0.035), while the proportions of other ILC subsets were comparable between the two groups (Fig. 2b). When we evaluated the CCR6+ ILC3/total ILC ratio in PB, PLNs, and joints of arthritic mice, we observed a higher relative ratio in LN than in PB (18.6% vs 6.2%, *p* = 0.00020) and a higher ratio in joints than in LN (29.6% vs 18.6%, *p* = 0.057), although the latter was not statistically significant (Fig. 2c). We interpret this gradual change as due to the induction of severe arthritis, as we observed no differences in the proportion of CCR6+ ILC3s to total ILCs in the joints and LN of pre-arthritic mice (16.7% vs 16.1%, *p* = 0.58) (Additional file 2: Figure S2). These data indicate that CCR6+ ILC3s may preferentially accumulate at local sites of inflammation.

### CCR6+ ILC3s produce high levels of IL-17A and IL-22 in arthritic mice

**Fig. 3:**
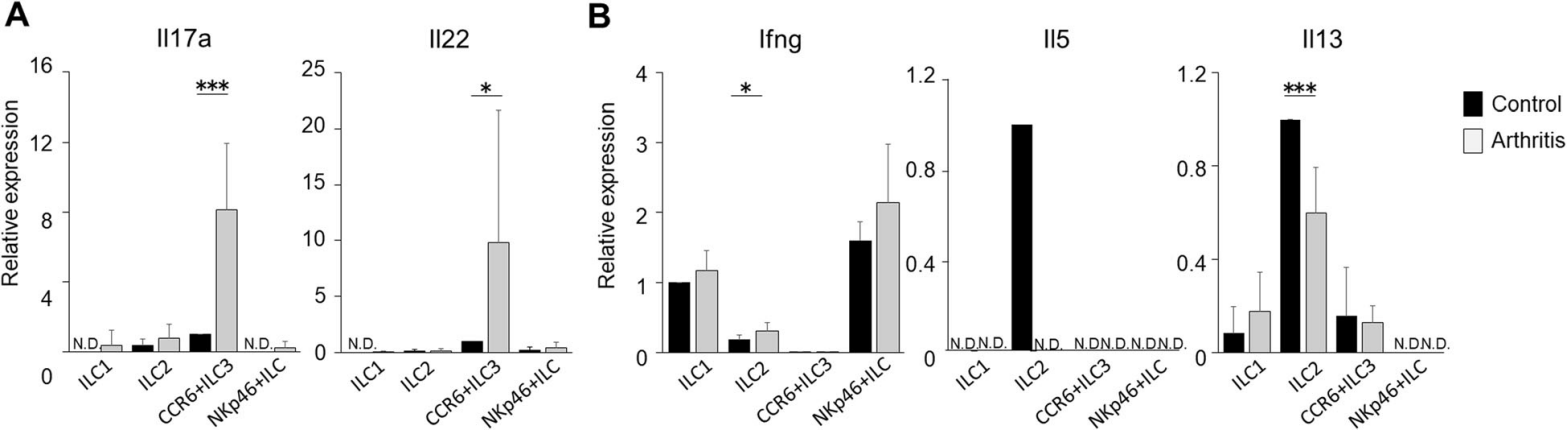
IL-17A and IL-22 mRNA levels increase significantly in arthritic mice. **a**, **b** Transcript levels of IL-17A and IL-22 (**a**) and IFN-γ, IL-5, and IL-13 (**b**) in ILC subsets of control and arthritic mice. CCR6+ ILC3s in mice the latter show elevated levels of IL-17A and IL-22 mRNA. Results are shown as means ± SD in triplicate samples of two to three independent experiments, each performed using two to three mice. In **a** and **b**, ILC subsets were obtained from mouse spleen cells. Cytokine mRNA expression was analyzed by quantitative PCR. Results are expressed as means ± SD in triplicate samples from two to three independent experiments performed using one to three mice. Results are expressed as relative expression to the unstimulated representative population which produced each cytokine. The analysis was conducted using the unpaired Student’s *t* test. (*p < 0.05, **p < 0.01, ***p < 0.001). N.S., not significant; N.D., not detected

Next, we compared cytokine mRNA levels in ILC subpopulations isolated from the spleen of control and arthritic mice. CCR6+ ILC3s in arthritic mice exhibited significantly higher expression levels of IL-17A and IL-22 mRNA relative to control mice (Fig. 3a). The level of IFN-γ transcripts in ILC1s was comparable in control and CIA mice. The expression of IL-5 mRNA was confirmed only in ILC2 of control mice, and the expression level of IL-13 mRNA was lower in arthritic mice than in control mice (Fig. 3b). These results suggest that the increase in cytokine production in CIA mice is specific to CCR6+ ILC3s.

### SF of patients with active RA shows increased numbers of CCR6+ ILCs

**Fig. 4:**
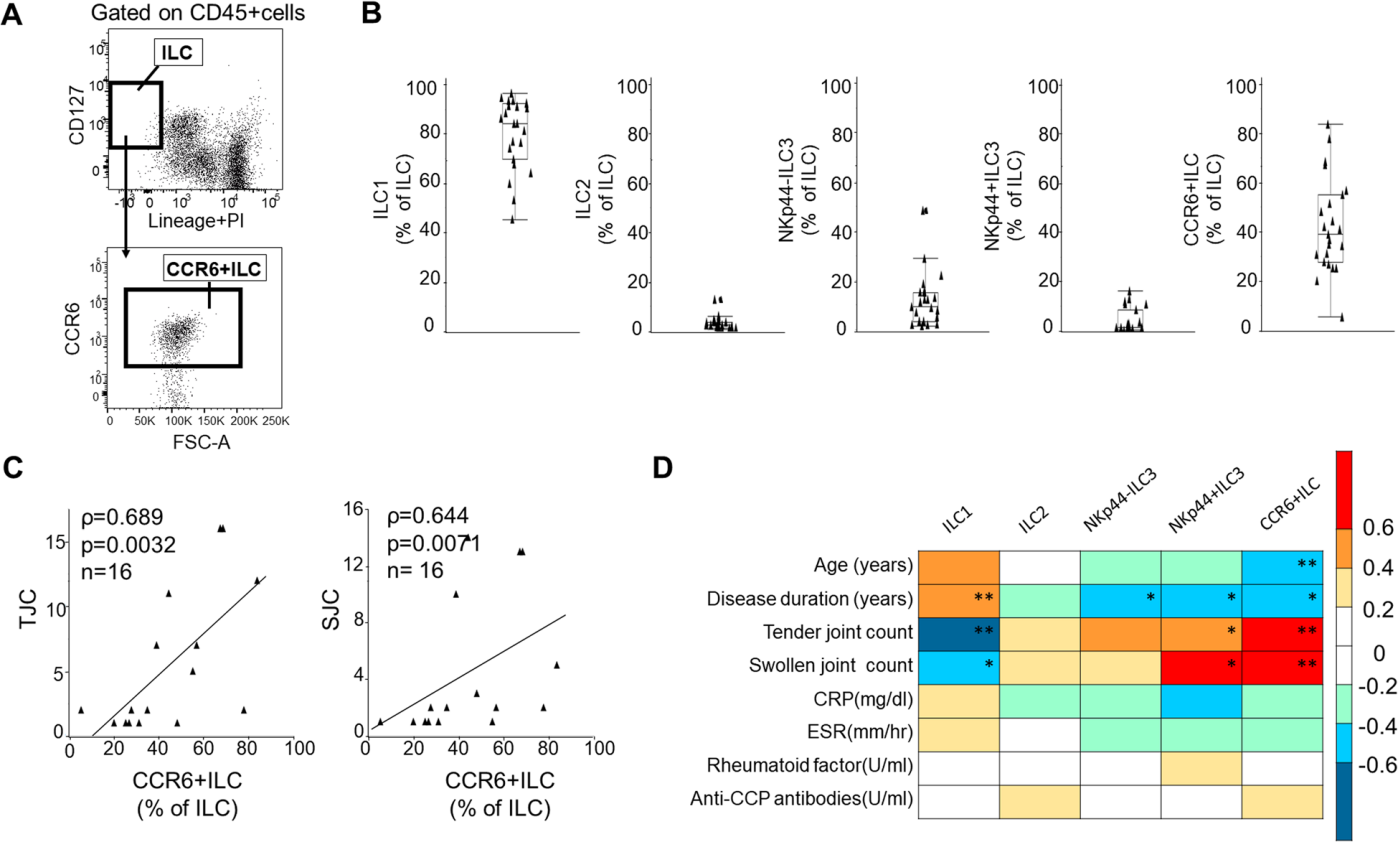
CCR6+ ILC/total ILC ratio in SF of RA patients is positively correlated with SJC and TJC. **a** Gating strategy for CCR6+ ILCs. ILCs were defined as CD45+, lineage−, and CD127+ cells. **b** Proportion of ILC1s, ILC2s, NKp44− ILC3s, NKp44+ ILC3s, and CCR6+ ILCs in SF of patients with RA. The proportion of CCR6+ ILCs relative to total ILCs varied widely. Results are expressed as the median and interquartile range and analyzed based on the Wilcoxon rank-sum test. (*p < 0.05, **p < 0.01, ***p < 0.001). **c** Correlation of the proportion of CCR6+ ILCs in SF and either tender joint count or swollen joint count. Data are presented as scatter plots with a linear fit and analyzed using Spearman’s rank correlation coefficient. **d** Heat map showing correlation coefficients between ILC subsets and various clinical parameters as analyzed by calculating Spearman’s rank correlation coefficient. The value of the axis shows a correlation coefficient. (*p < 0.05, **p < 0.01, ***p < 0.001)

We next extended our analysis to human RA. To determine whether the proportion of CCR6+ ILCs to total ILCs increases in SF of RA patients as it does in the joints of CIA mice, we analyzed ILC subsets in 23 samples of RA SF. To examine whether CCR6 could be a marker of disease activity, we evaluate CCR6-positive cells in ILCs separately from the other ILC subsets (Fig. 4a). The other subsets, ILC1, ILC2, NKp44− ILC3, and NKp44+ ILC3, were evaluated using methods previously established [21] (Additional file 3: Figure S3A). CCR6+ ILCs in human peripheral blood express RORC, IL-17A, and IL-22 mRNA (Additional file 4: Figure S4) and produce these cytokines after stimulation (Additional file 5: Figure S5). The proportion of CCR6+ ILCs among total ILCs in SF of RA patients ranged widely from 5.36 to 83.6%, while the proportions of other subsets in patients showed less variation (Fig. 4b).

To assess the characteristics of patients exhibiting a high proportion of CCR6+ ILCs, we examined the potential correlation of the proportion of each ILC subset in SF with clinical parameters by calculating Spearman’s correlation coefficients. Clinical parameters assessed were age at examination, disease duration, swollen joint count (SJC), tender joint count (TJC), C-reactive protein (CRP) levels (mg/dl), erythrocyte sedimentation rate (ESR) (mm/h), rheumatoid factor (RF) levels (U/ml), and anti-CCP antibody levels (U/ml) (Additional file 9 Table S3). We excluded sex from the analysis, as there were only three male patients. We found that the proportion of CCR6+ ILCs increased with TJC (*ρ*=0.689, *p* = 0,0032) and with SJC (*ρ*=0.644, *p* = 0.0071) (Fig. 4c).

Relevant to other ILC subsets, ILC1 proportions were negatively correlated with TJC and SJC, while ILC2s numbers were not significantly correlated with clinical parameters. The proportion of NKp44+ ILC3s was positively correlated with TJC and SJC (Fig. 4d and Additional file 9: Table S3). The correlation of NKp44+ ILCs with SJC and TJC may reflect the expression of CCR6 protein on their surface because almost all NKp44+ ILC3s expressed CCR6 protein (Additional file 3: Figure S3B). Taken together, these results suggest that the presence of CCR6+ ILCs exacerbates joint inflammation in patients with RA.

### CCR6+ ILCs migrate to the joints in parallel with increasing CCL20 levels in SF of RA patients

**Fig. 5:**
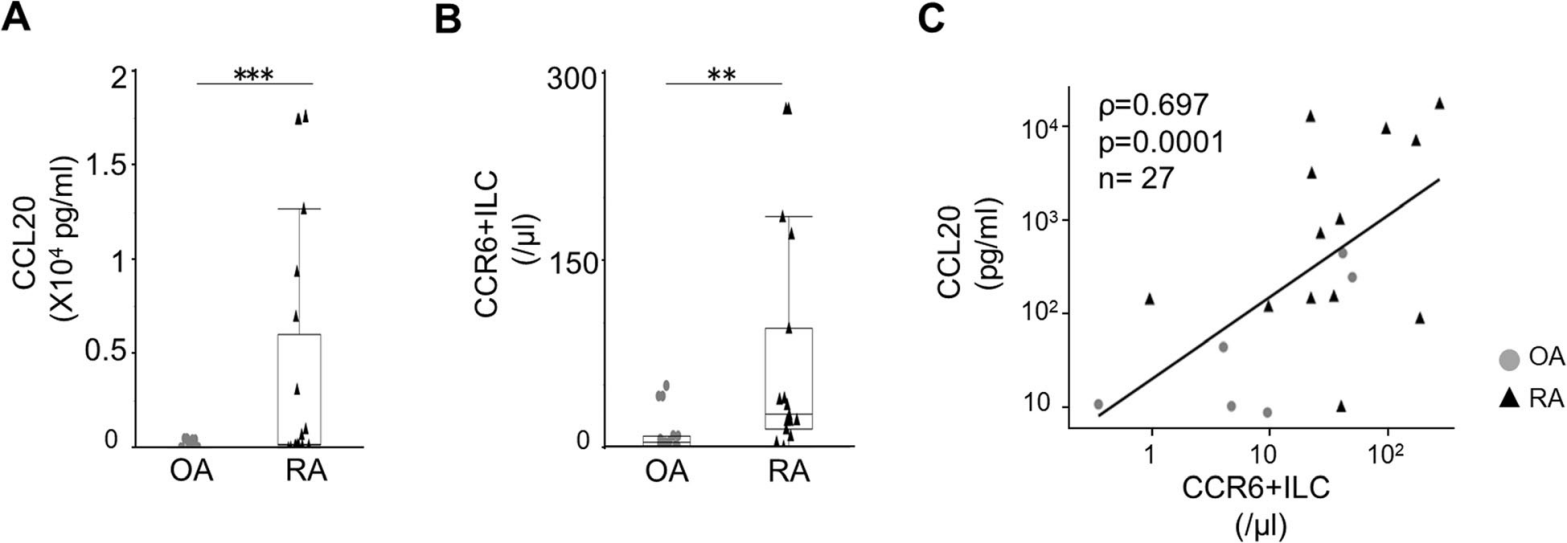
CCR6+ ILC number is positively correlated with SF CCL20 levels. **a** Comparison of CCL20 concentration in SF of patients with OA (*n* = 24) and RA (*n* = 16). CCL20 concentration in SF of patients with RA was significantly higher than that in SF from OA patients. CCL20 levels in synovial fluid were measured by ELISA. Data was analyzed using the Wilcoxon rank-sum test. (*p < 0.05, **p < 0.01, ***p < 0.001). **b** The absolute number of CCR6+ ILCs in SF of patients of OA (*n* = 13) and RA (*n* = 15). Data was analyzed using the Wilcoxon rank-sum test. (*p < 0.05, **p < 0.01, ***p < 0.001). **c** Correlation of the number of CCR6+ ILCs and CCL20 concentration in SF of patients with OA (*n* = 13) and RA (*n* = 15). The number of CCR6+ ILCs was positively correlated with CCL20 concentration. Data are presented as scatter plots with a linear fit and analyzed by calculating Spearman’s rank correlation coefficient

The CCR6 ligand CCL20 controls the migration of CCR6+ cells [22]. Thus, we evaluated CCL20 levels in SF of 14 RA and in 13 control OA patients. The latter showed barely detectable CCL20 levels, while SF from RA patients showed high CCL20 levels, as previously reported [23] (Fig. 5a). Next, we examined the relationships between CCL20 concentration and absolute numbers of CCR6+ ILCs in SF. As shown in Fig. 5b, c, the absolute number of CCR6+ ILCs was significantly higher in SF of RA patients and CCL20 levels increased in parallel with the absolute number of CCR6+ ILCs.

## Discussion

In this study, we evaluated the roles of ILCs in RA development using model mice and human patients. In CIA mice, CCR6+ ILC3s accumulated in the joints and exhibited high expression of mRNAs encoding the cytokines IL-17A and IL-22. In humans, the number of CCR6+ ILCs increased in SF of patients with active RA and accumulated in inflamed joints in parallel with CCL20 concentration in SF. These results suggest that CCR6+ ILC3s have some roles in the development of RA.

To date, there has been no consensus about which ILC subset has the greatest impact on RA progression. Hirota et al. showed that GM-CSF-producing ILC2s had a pathogenic role in the development of arthritis [13], although recent studies reported that ILC2s had a protective role [11, 12]. Zhu et al. reported that the number of CD3− CD56+ NKp44+ cells, which are ILC3-like cells, increases in SF and PB of patients with RA and that those numbers are positively correlated with disease activity [15, 16]. On the other hand, a subset of NK cells, which are ILC1-like cells, was identified in SF of patients with inflammatory arthritis including RA, spondyloarthropathy, and juvenile rheumatoid arthritis [14]. Leijten et al. also demonstrated that ILC1s predominate in RA SF [7], and Rodríguez-Carrio et al. revealed that the frequency of ILC1s in LN biopsy was significantly increased in RA patients [24]. However, in our hands, the ILC1 subpopulation did not increase in the joints of CIA model mice nor were Th1 cytokine expression levels elevated after arthritis induction. Furthermore, although ILC1s were the predominant population in SF of RA patients, their proportion to total ILCs was negatively correlated with the number of inflamed joints, suggesting that a different cell type is a critical factor in RA inflammation.

In this study, we observed differences in ILC phenotypes between mice and humans. The proportion of CCR6+ ILC3s in the joints of CIA mice increased in almost all cases, while there was considerable variability in the proportion of CCR6+ ILCs between SF samples from RA patients (Figs. 2b and 4b). It is largely recognized that IL-17 signaling plays a key role in inflammatory arthritis in mouse models [25], while in humans, IL-17 signaling does not always play a critical role in inflammation associated with RA. Several anti-IL-17 therapies for RA have not demonstrated sufficient clinical improvements [26, 27], while meta-analyses show favorable results for ACR20 or ACR50 improvement [28, 29]. These results indicate that the relative importance of Th17 pathways differs among patients or at different disease stages. Accordingly, it is crucial to know which patients with RA will respond well to IL-17 blockade therapy. Identification of a surrogate marker for a high proportion of CCR6+ ILCs in SF might help stratify RA patients as candidates for anti-IL-17 therapy.

The limitation of our study was that we could not evaluate the correlations between CCR6+ ILCs and composite measures of RA disease activity, such as Disease Activity Score 28 (DAS28)-CRP or DAS28-ESR, due to unavailability of appropriate data. Secondly, the sample size of both mice and human studies was small. Lastly, we could not clearly identify ILCs in the synovium, the site of inflammation in RA. Though we detected ILC subsets in the synovium obtained from two patients with RA by flow cytometry (Additional file 6: Figure S6), the data was unsuitable to discuss because the sample size was too small. We also tried to identify ILCs in the joints of wild-type or CIA mice by immunohistochemistry, but we failed presumably due to technical problems. Recently, Noort et al. showed that ILC3s, which were defined as CD3− RORγt+ cells, were present in human synovium by immunohistochemistry [30]. Zhang et al. reported that recent advances at single-cell analysis, such as mass cytometry and RNA sequencing, contributed to identify new cell population in synovium that might be related to the pathogenesis of RA [31]. To provide more information about the role of ILCs in RA development, we should collect a large number of samples and evaluate ILCs in synovium by these new techniques.

In conclusion, the present study demonstrates that CCR6+ ILC3s are enriched in the inflamed joints of CIA mice and active RA patients and could play an important role in RA development.

## Additional files

Additional file 1: **Figure S1.** Type II collagen specific IgG is detected in sera from pre-arthritic and arthritic mice. Levels of anti-CIIIgG antibodies in sera of control (*n*=9), pre-arthritic (*n*=15) and arthritic (*n*=12) mice. Antibody levels are expressed as optical density (O.D.) at 450 nm, as measured by ELISA. Marks in graphs represent data from individual mice. (Kruskal Wallis test; *p* <0.0001) Results are expressed as the median and interquartile range based on the Wilcoxon rank-sum test. (*p<0.05, **p<0.01, ***p<0.001) (TIF 61 kb)

Additional file 2: **Figure S2.** CCR6+ ILC3/total ILC ratio in joints of pre-arthritic mice is comparable to that in PLNs. The proportion of CCR6+ ILC3s to ILCs in peripheral blood (*n* = 8), popliteal lymph nodes (*n* = 5) of control mice (left). The proportion of CCR6+ ILC3s to ILCs in peripheral blood (*n* = 13), popliteal lymph nodes (*n* = 10) and joints (n = 5) of pre-arthritic mice (right). Marks in graphs represent data points from individual mice. (Kruskal Wallis test; *p* = 0.0017) Results are expressed as the median and interquartile range and analyzed using the Wilcoxon rank-sum test. (*p<0.05, **p<0.01, ***p<0.001). N.S., not significant; N.D., not detected. (TIF 65 kb)

Additional file 3: **Figure S3.** ILC subsets in SF of RA patients. A. Gating strategy for ILCs from SF of a patient with RA. ILCs were defined as CD45+, lineage -, CD127+ cells. ILC1 cells: CRTH2- CD117- NKp44-; ILC2 cells: CRTH2+; NKp44- ILC3 cells: CRTH2-CD117+ NKp44-; and NKp44+ ILC3 cells: CRTH2- CD117+ NKp44+ (delete). B. Representative FACS plots of CCR6+ and NKp44+ ILCs from SF of patients with RA. These are gated on ILCs shown at left in panel A. (TIF 128 kb)

Additional file 4: **Figure S4.** Expression of RORC and Th17 cytokines mRNA in human CCR6+ ILCs. A. CCR6+ ILCs in human peripheral blood expresses RORC in the steady state. Results are shown as means ± SD in triplicate samples of four patients. B. CCR6+ ILCs in human peripheral blood expresses IL-17A and IL-22 mRNA after stimulation with PMA/ ionomycin. Results are shown as means ± SD in triplicate samples of two patients. (TIF 79 kb)

Additional file 5: **Figure S5.** Production of Th17 cytokines in human CCR6+ ILCs and Th subsets. A.B. CCR6+ ILCs in human peripheral blood produce Th17 cytokines after stimulation with PMA/ ionomycin for five hours. Representative data of the production of IL-17 (A) and IL-22 (B) by CCR6+ ILCs. C.D. Representative data of the production of IL-17 (C) and IL-22 (D) by Th1, Th2, Th17 and Th1/17 after stimulation with PMA/ ionomycin for five hours. Th subsets were defined as single cells within the lymphocyte gate on the scatter plot that were CD3 and CD4 positive. Th1, Th2, Th17 and Th1/17 were defined as CXCR3+ CCR6-, CXCR3- CCR6-, CXCR3- CCR6+ and CXCR3+ CCR6+ respectively. (TIF 369 kb)

Additional file 6: **Figure S6.** ILC subsets in synovium of patients with RA. FACS plots of ILC subsets in synovium of patients with RA. (TIF 189 kb)

Additional file 7: **Table S1.** List of antibodies used in flow cytometry (DOCX 23 kb)

Additional file 8: **Table S2.** Clinical characteristics of patients with RA. TCZ, Tocilizumab. ETN, Etanercept. MZR, Mizoribine. IGU, Iguratimod. (DOCX 16 kb)

Additional file 9: **Table S3.** Correlation of clinical parameters with the proportion of ILC subsets in total ILCs based on the Spearman’s rank correlation coefficient. (*p<0.05, **p<0.01, ***p<0.001). (TIF 192 kb)

## Data Availability

All data generated or analyzed during this study are included in this published article and its supplementary information files.

## Abbreviations

ILCs: Innate lymphoid cells
RA: Rheumatoid arthritis
CIA: Collagen-induced arthritis
PB: Peripheral blood
LNs: Lymph nodes
SF: Synovial fluid
TJC: Tender joint count
SJC: Swollen joint count
CCL20: CC chemokine ligand 20
T-bet: T-box transcription factor
IFN-γ: Interferon-γ
GATA3: GATA-binding protein 3
IL-4: Interleukin-4
RORγt: Retinoic acid receptor-related orphan receptor γt
CCR6: CC chemokine receptor 6
NCR: Natural cytotoxicity receptor
NK: Natural killer
SpA: Spondyloarthritis
CII: Type II collagen
CFA: Complete Freund’s adjuvant
ELISA: Enzyme-linked immunosorbent assay
OA: Osteoarthritis
CRTH2: Chemoattractant receptor homologous molecule expressed on Th2 cells
TFs: Transcription factors
Tbx21: T-box 21
RORC: RAR-related orphan receptor C
PLNs: Popliteal lymph nodes
CRP: C-reactive protein
ESR: Erythrocyte sedimentation rate
RF: Rheumatoid factor
DAS28: Disease Activity Score 28
