# Supplementary figures and images for: CCR6+ group 3 innate lymphoid cells accumulate in inflamed joints in rheumatoid arthritis and produce Th17 cytokines
Source: Arthritis Res Ther. 2019 Aug 30;21:198. doi: 10.1186/s13075-019-1984-x (PMC6716915; doi:10.1186/s13075-019-1984-x)

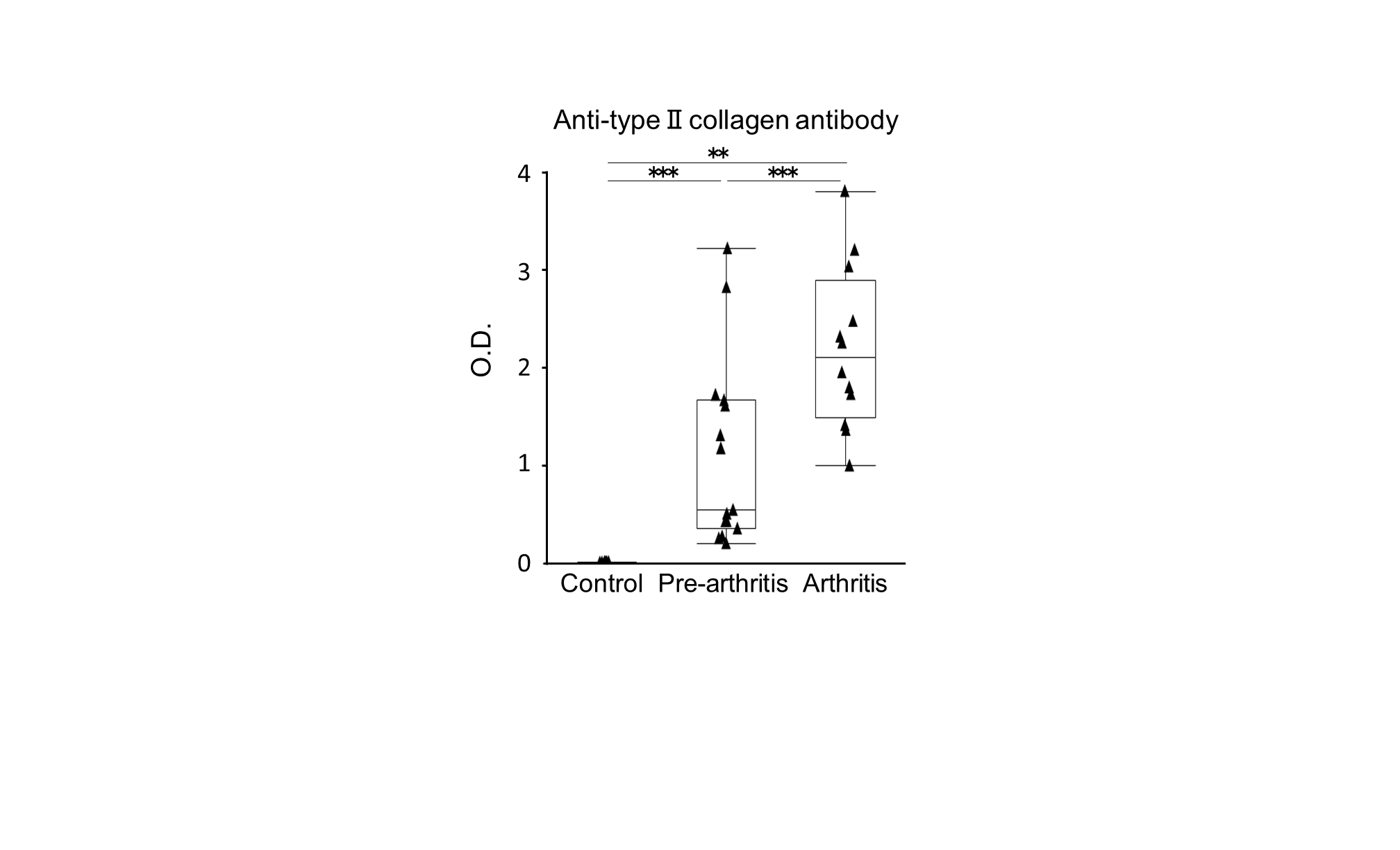

Supplement: Supplementary file 1 — Figure S1. Type II collagen specific IgG is detected in sera from pre-arthritic and arthritic mice. Levels of anti-CIIIgG antibodies in sera of control (n=9), pre-arthritic (n=15) and arthritic (n=12) mice. Antibody levels are expressed as optical density (O.D.) at 450 nm, as measured by ELISA. Marks in graphs represent data from individual mice. (Kruskal Wallis test; p <0.0001) Results are expressed as the median and interquartile range based on the Wilcoxon rank-sum test. (*p<0.05, **p<0.01, ***p<0.001) (TIF 61 kb) [file 13075_2019_1984_MOESM1_ESM.tif]

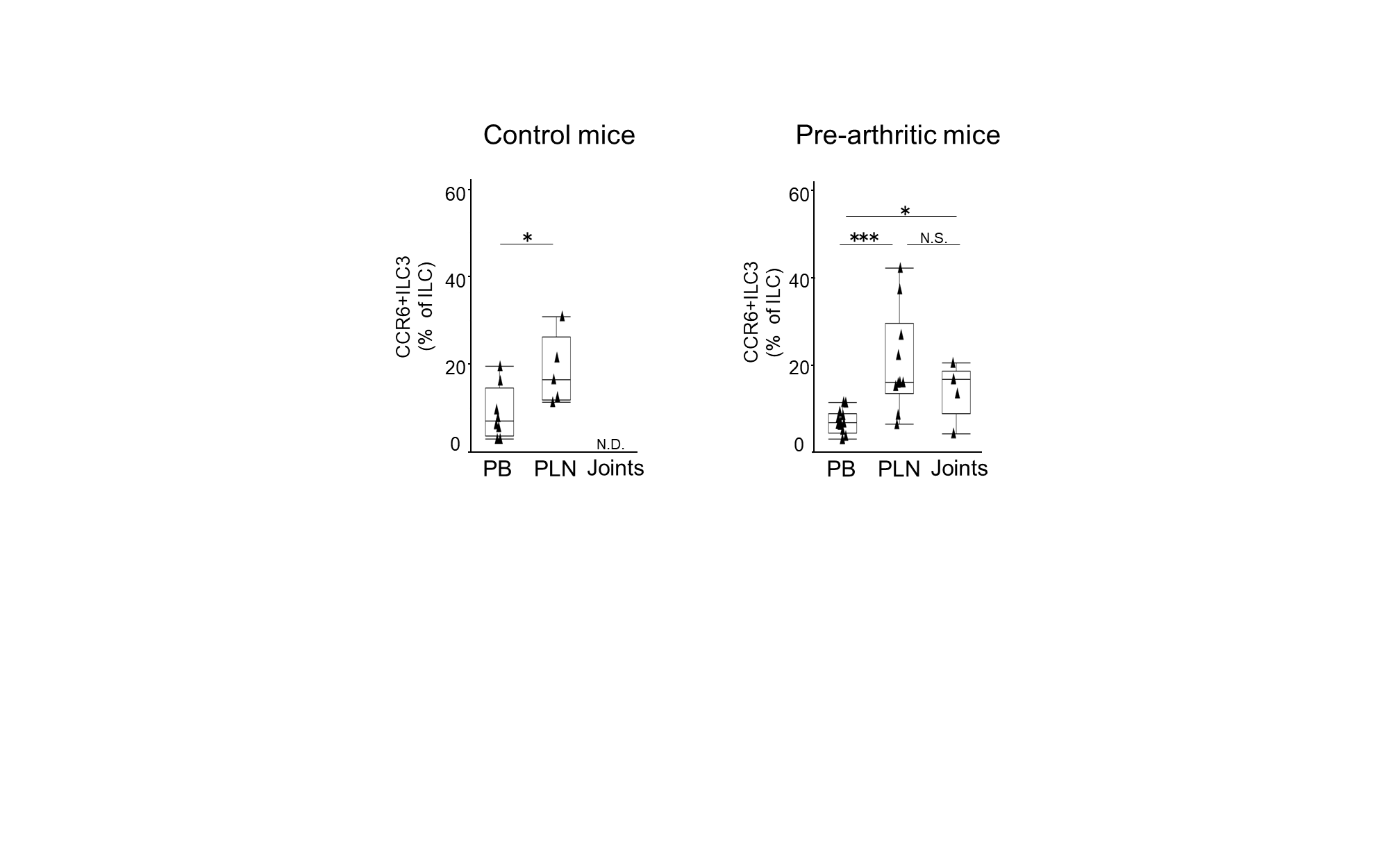

Supplement: Supplementary file 2 — Figure S2. CCR6+ ILC3/total ILC ratio in joints of pre-arthritic mice is comparable to that in PLNs. The proportion of CCR6+ ILC3s to ILCs in peripheral blood (n = 8), popliteal lymph nodes (n = 5) of control mice (left). The proportion of CCR6+ ILC3s to ILCs in peripheral blood (n = 13), popliteal lymph nodes (n = 10) and joints (n = 5) of pre-arthritic mice (right). Marks in graphs represent data points from individual mice. (Kruskal Wallis test; p = 0.0017) Results are expressed as the median and interquartile range and analyzed using the Wilcoxon rank-sum test. (*p<0.05, **p<0.01, ***p<0.001). N.S., not significant; N.D., not detected. (TIF 65 kb) [file 13075_2019_1984_MOESM2_ESM.tif]

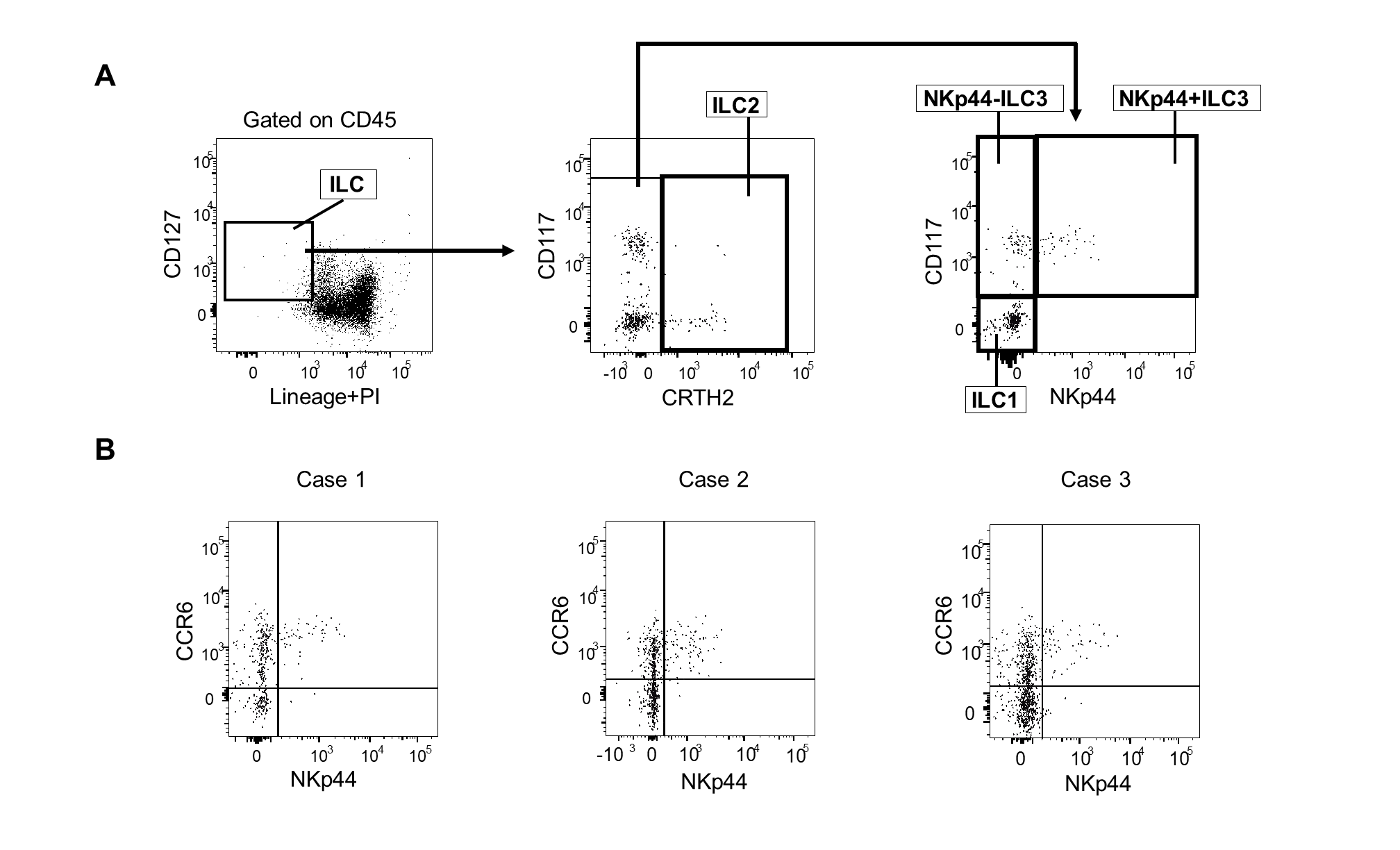

Supplement: Supplementary file 3 — Figure S3. ILC subsets in SF of RA patients. A. Gating strategy for ILCs from SF of a patient with RA. ILCs were defined as CD45+, lineage -, CD127+ cells. ILC1 cells: CRTH2- CD117- NKp44-; ILC2 cells: CRTH2+; NKp44- ILC3 cells: CRTH2-CD117+ NKp44-; and NKp44+ ILC3 cells: CRTH2- CD117+ NKp44+ (delete). B. Representative FACS plots of CCR6+ and NKp44+ ILCs from SF of patients with RA. These are gated on ILCs shown at left in panel A. (TIF 128 kb) [file 13075_2019_1984_MOESM3_ESM.tif]

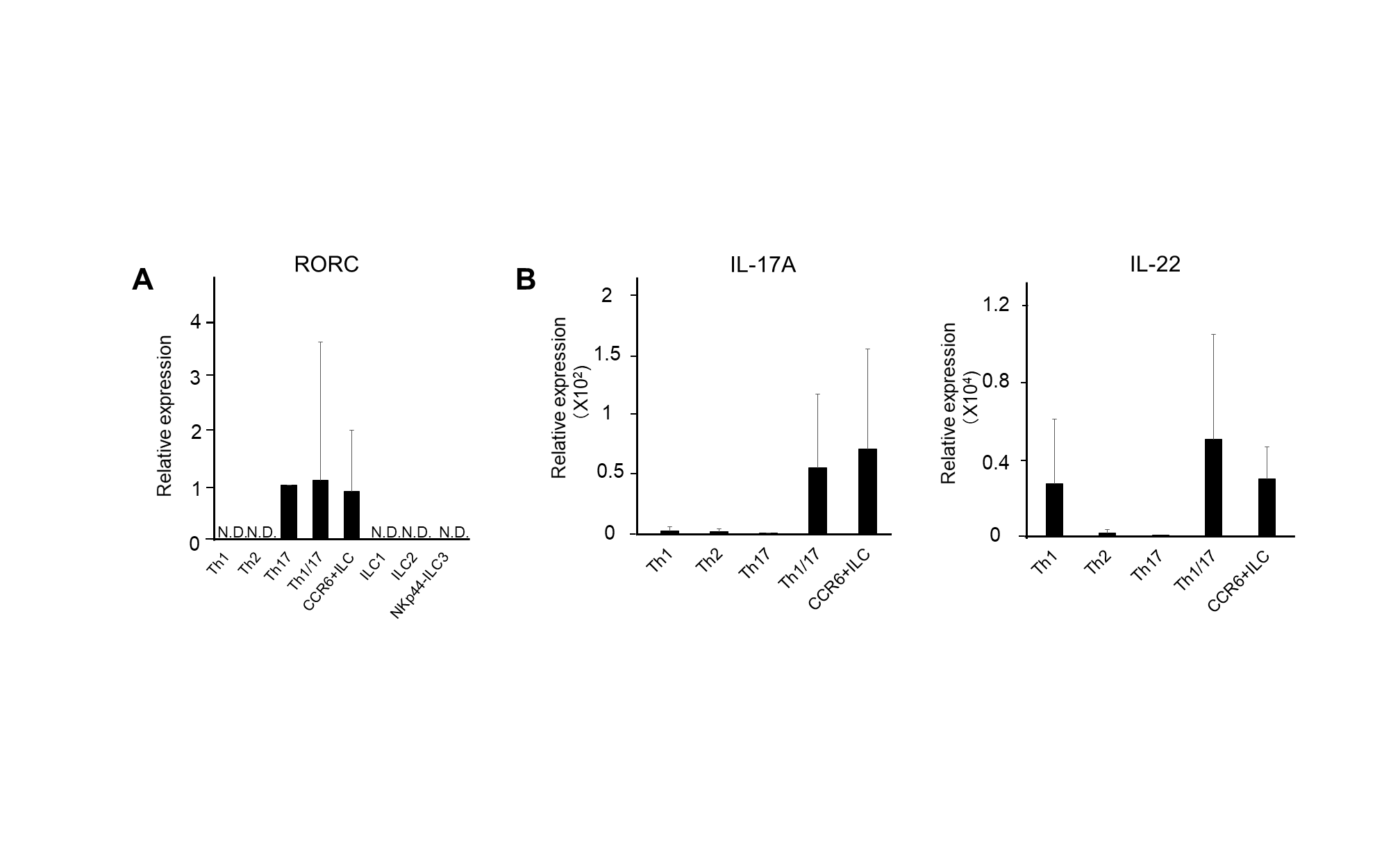

Supplement: Supplementary file 4 — Figure S4. Expression of RORC and Th17 cytokines mRNA in human CCR6+ ILCs. A. CCR6+ ILCs in human peripheral blood expresses RORC in the steady state. Results are shown as means ± SD in triplicate samples of four patients. B. CCR6+ ILCs in human peripheral blood expresses IL-17A and IL-22 mRNA after stimulation with PMA/ ionomycin. Results are shown as means ± SD in triplicate samples of two patients. (TIF 79 kb) [file 13075_2019_1984_MOESM4_ESM.tif]

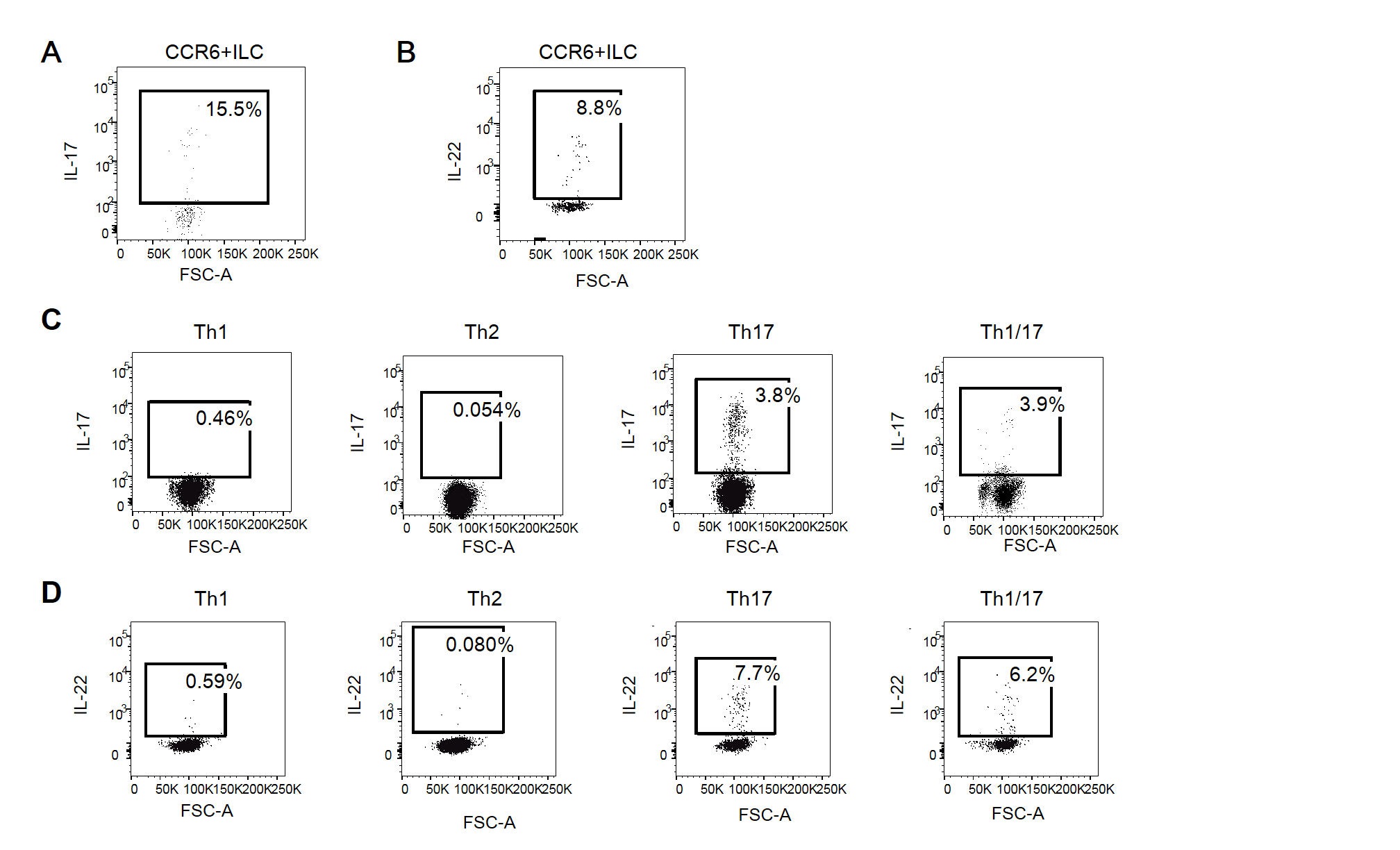

Supplement: Supplementary file 5 — Figure S5. Production of Th17 cytokines in human CCR6+ ILCs and Th subsets. A.B. CCR6+ ILCs in human peripheral blood produce Th17 cytokines after stimulation with PMA/ ionomycin for five hours. Representative data of the production of IL-17 (A) and IL-22 (B) by CCR6+ ILCs. C.D. Representative data of the production of IL-17 (C) and IL-22 (D) by Th1, Th2, Th17 and Th1/17 after stimulation with PMA/ ionomycin for five hours. Th subsets were defined as single cells within the lymphocyte gate on the scatter plot that were CD3 and CD4 positive. Th1, Th2, Th17 and Th1/17 were defined as CXCR3+ CCR6-, CXCR3- CCR6-, CXCR3- CCR6+ and CXCR3+ CCR6+ respectively. (TIF 369 kb) [file 13075_2019_1984_MOESM5_ESM.tif]

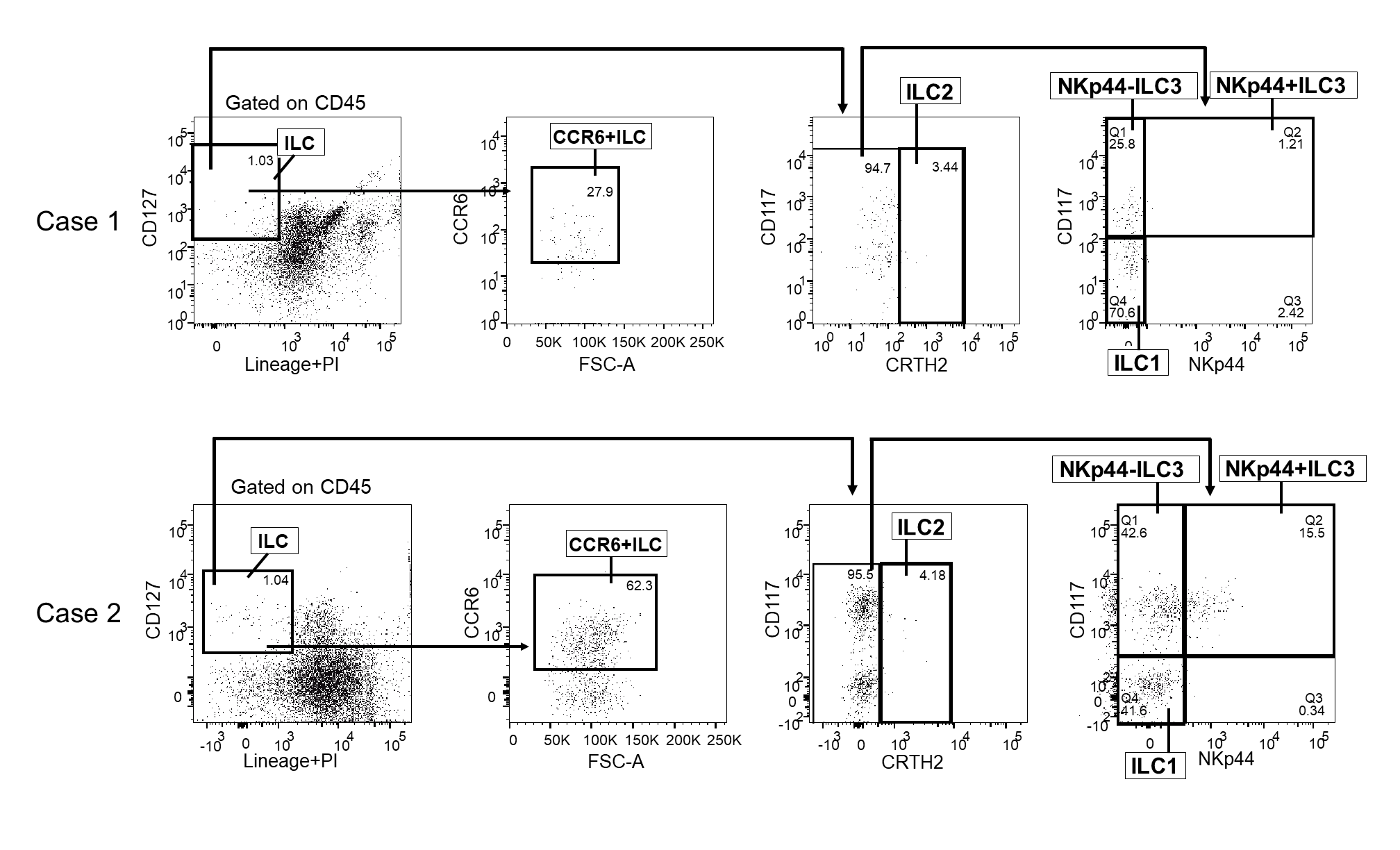

Supplement: Supplementary file 6 — Figure S6. ILC subsets in synovium of patients with RA. FACS plots of ILC subsets in synovium of patients with RA. (TIF 189 kb) [file 13075_2019_1984_MOESM6_ESM.tif]

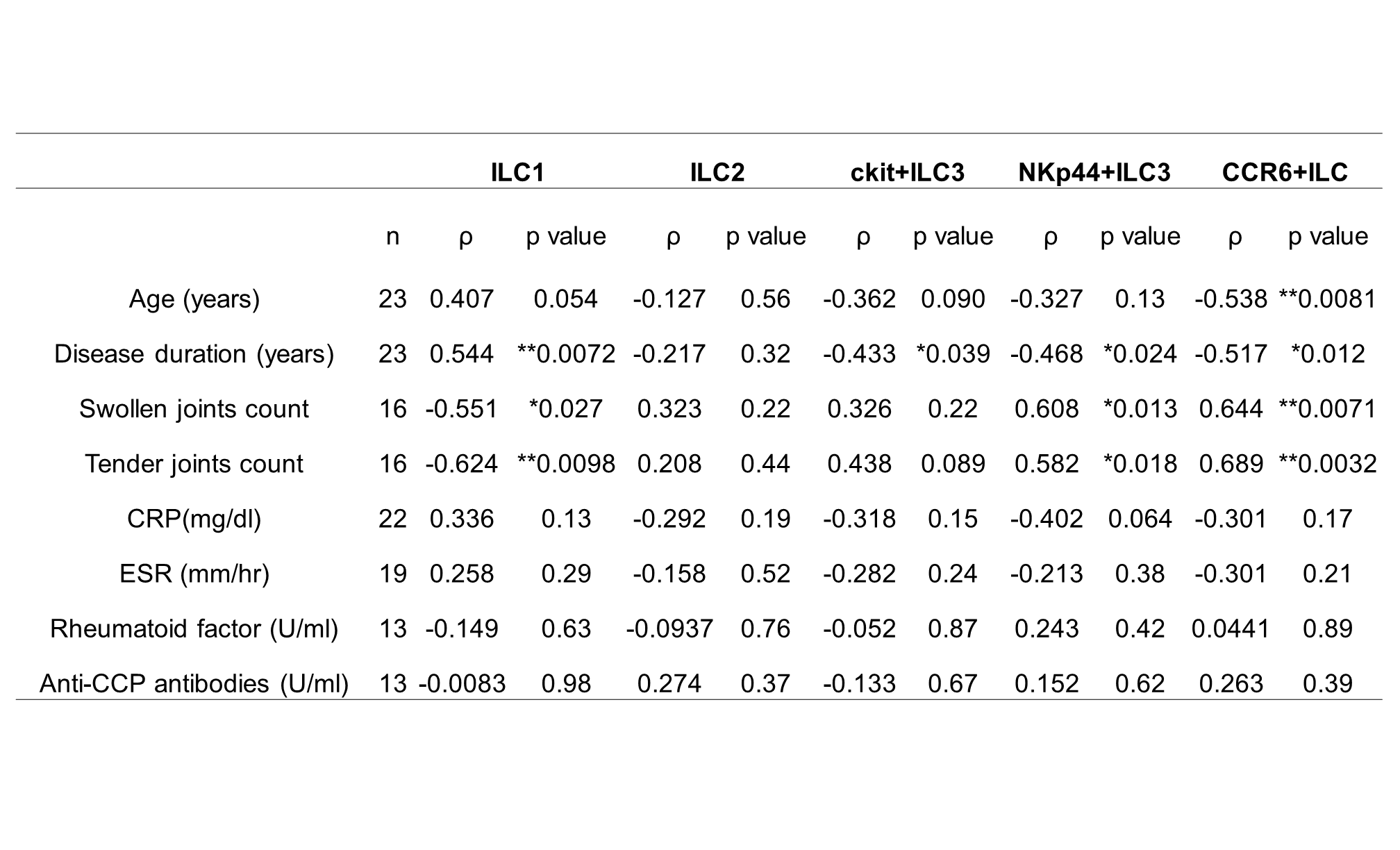

Supplement: Supplementary file 9 — Table S3. Correlation of clinical parameters with the proportion of ILC subsets in total ILCs based on the Spearman’s rank correlation coefficient. (*p<0.05, **p<0.01, ***p<0.001). (TIF 192 kb) [file 13075_2019_1984_MOESM9_ESM.tif]
