# Supplementary material for: CCR6+ group 3 innate lymphoid cells accumulate in inflamed joints in rheumatoid arthritis and produce Th17 cytokines
Source: Arthritis Res Ther. 2019 Aug 30;21:198. doi: 10.1186/s13075-019-1984-x (PMC6716915; doi:10.1186/s13075-019-1984-x)
Supplement: Supplementary file 7 — Table S1. List of antibodies used in flow cytometry (DOCX 23 kb) [file 13075_2019_1984_MOESM7_ESM.docx]

Additional File 7: Supplementary Table 1.

|  | Target Antigen | | Clone | Conjugate | Company | Catalog# |
| --- | --- | --- | --- | --- | --- | --- |
| Mouse | CD3 |  | 17A2 | PerCP-Cy5.5 | BioLegend | 100218 |
|  | CD8a |  | 53-6.7 | PerCP-Cy5.5 | BD Biosciences | 551162 |
|  | CD19 |  | 1D3 | PerCP-Cy5.5 | BD Biosciences | 561113 |
|  | CD45R/B220 |  | RA3-6B2 | PerCP-Cy5.5 | BD Biosciences | 552771 |
|  | Ter119 |  | TER-119 | PerCP-Cy5.5 | BioLegend | 116228 |
|  | Ly-6G |  | 1A8 | PerCP-Cy5.5 | BD Biosciences | 560602 |
|  | CD11c |  | HL3 | PerCP-Cy5.5 | BD Biosciences | 560584 |
|  | CD11b |  | M1/70 | PerCP-Cy5.5 | BD Biosciences | 55093 |
|  | CD45.2 |  | 104 | PE/Cy7 | BioLegend | 109830 |
|  | CD90.2 | (Thy1.2) | 53-2.1 | BV510 | BioLegend | 140319 |
|  | CD127 | (IL-7R) | A7R34 | BV421 | BioLegend | 135027 |
|  | NK1.1 |  | PK136 | APC | BioLegend | 108710 |
|  | T1/ST2 | (IL-33R) | DJ8 | biotin | MD Bioproducts | 101001B |
|  | streptavidin |  |  | APC/Cy7 | BioLegend | 405208 |
|  | CD196 | (CCR6) | 29-2L17 | PE | BioLegend | 129804 |
|  | CD335 | (NKp46) | 29A1.4 | FITC | BioLegend | 137606 |
| Human | CD3 |  | UCHT1 | PerCP-Cy5.5 | BioLegend | 300430 |
|  | CD19 |  | HIB19 | PerCP-Cy5.5 | BioLegend | 302230 |
|  | CD16 |  | 3G8 | PerCP-Cy5.5 | BioLegend | 302028 |
|  | CD94 |  | HP-3D9 | PerCP-Cy5.5 | BD Biosciences | 562361 |
|  | CD11c |  | Bu15 | PerCP-Cy5.6 | BioLegend | 337210 |
|  | CD11b |  | M1/70 | PerCP-Cy5.7 | BioLegend | 101228 |
|  | CD14 |  | 63D3 | PerCP-Cy5.5 | BioLegend | 367110 |
|  | CD1c |  | F10/21A3 | PerCP-Cy5.5 | BD Biosciences | 565424 |
|  | CD235ab |  | HIR2 | PerCP-Cy5.5 | BioLegend | 306614 |
|  | FcεRⅠ |  | AER-37(CRA-1) | PerCP-Cy5.5 | BioLegend | 334621 |
|  | CD34 |  | 8G12 | PerCP-Cy5.5 | BD Biosciences | 347203 |
|  | CD117 | (c-Kit) | 104D2 | BV421 | BD Biosciences | 563856 |
|  | CD294 | (CRTH2) | BM16 | Alexa Fluor647 | BD Biosciences | 561797 |
|  | CD336 | (NKp44) | p44-8.1 | PE | BD Biosciences | 558563 |
|  | CD196 | (CCR6) | G034E3 | APC/Cy7 | BioLegend | 353432 |
|  | CD45 |  | Hi30 | BV510 | BD Biosciences | 563204 |
|  | CD127 | (IL-7R) | R34.34 | PE/Cy7 | BECKMAN COULTER | A64618 |
