# Supplementary material for: CCR6+ group 3 innate lymphoid cells accumulate in inflamed joints in rheumatoid arthritis and produce Th17 cytokines
Source: Arthritis Res Ther. 2019 Aug 30;21:198. doi: 10.1186/s13075-019-1984-x (PMC6716915; doi:10.1186/s13075-019-1984-x)
Supplement: Supplementary file 8 — Table S2. Clinical characteristics of patients with RA. TCZ, Tocilizumab. ETN, Etanercept. MZR, Mizoribine. IGU, Iguratimod. (DOCX 16 kb) [file 13075_2019_1984_MOESM8_ESM.docx]

|  | N | Median | range |
| --- | --- | --- | --- |
| Age | 17 | 66 | 52-79 |
| Disease duration  (years) | 17 | 9.4 | 1-33 |
| Swollen joints count | 14 | 2 | 1-14 |
| Tender joints count | 14 | 2 | 1-16 |
| CRP(mg/dl) | 16 | 0.87 | 0.02-6.73 |
| ESR(mm/hr) | 16 | 26 | 3-93 |
| PSL(mg/day) | 15 | 3 | 0-7 |
| MTX(mg/week) | 16 | 6 | 0-12 |
| Biologic agent | 5  (TCZ 3, ETN2) |  |  |
| DMARDs | 2  (MZR 1, IGU1) |  |  |
| Rheumatoid factor (U/ml)  (positive(n)) | 17 | 32.5  (13) | 0-1231 |
| Anti-CCP antibody(U/ml)  (positive(n)) | 17 | 308  (10) | 1-4030 |
| sex | male(No.)  3 | male(%)  17.6 |  |

Additional File 8: Supplementary Table 2
